# Nanofibers-Based Piezoelectric Energy Harvester for Self-Powered Wearable Technologies

**DOI:** 10.3390/polym12112697

**Published:** 2020-11-16

**Authors:** Fatemeh Mokhtari, Mahnaz Shamshirsaz, Masoud Latifi, Javad Foroughi

**Affiliations:** 1 Textile Excellence & Research Centers, Textile Engineering Department, Amirkabir University of Technology, Tehran 1591634311, Iran; fm129@uowmail.edu.au (F.M.); latifi@aut.ac.ir (M.L.); 2 Intelligent Polymer Research Institute, University of Wollongong, Wollongong 2500, Australia; 3 New Technologies Research Center, Amirkabir University of Technology, Tehran 1591634311, Iran; shamshir@aut.ac.ir; 4 School of Electrical, Computer and Telecommunications Engineering, Faculty of Engineering and Information Sciences, University of Wollongong, Wollongong 2522, Australia; 5 Westgerman Heart and Vascular Center, University of Duisburg-Essen, 45122 Essen, Germany

**Keywords:** electrospinning, PVDF/LiCl nanofiber, piezoelectric nanogenerator, temperature, electrospun web thickness

## Abstract

The demands for wearable technologies continue to grow and novel approaches for powering these devices are being enabled by the advent of new energy materials and novel manufacturing strategies. In addition, decreasing the energy consumption of portable electronic devices has created a huge demand for the development of cost-effective and environment friendly alternate energy sources. Energy harvesting materials including piezoelectric polymer with its special properties make this demand possible. Herein, we develop a flexible and lightweight nanogenerator package based on polyvinyledene fluoride (PVDF)/LiCl electrospun nanofibers. The piezoelectric performance of the developed nanogenator is investigated to evaluate effect of the thickness of the as-spun mat on the output voltage using a vibration and impact test. It is found that the output voltage increases from 1.3 V to 5 V by adding LiCl as additive into the spinning solution compared with pure PVDF. The prepared PVDF/LiCl nanogenerator is able to generate voltage and current output of 3 V and 0.5 μA with a power density output of 0.3 μW cm^−2^ at the frequency of 200 Hz. It is found also that the developed nanogenerator can be utilized as a sensor to measure temperature changes from 30 °C to 90 °C under static pressure. The developed electrospun temperature sensor showed sensitivity of 0.16%/°C under 100 Pa pressure and 0.06%/°C under 220 Pa pressure. The obtained results suggested the developed energy harvesting textiles have promising applications for various wearable self-powered electrical devices and systems.

## 1. Introduction

Devices that harvest energy from ambient sources, such as solar, wind, mechanical vibrations, or thermal energy, have been developed recently as alternatives to fossil fuels. The idea of using renewable energy was raised several decades ago. However, unsustainable energy is still the main type consumed to an extent because of the constraints related to the climate or geography [1]. Among these, nanogenerators offer promise as a renewable source of energy that converts vibrational or mechanical energy directly into electrical energy [2,3]. As a piezoelectric polymer, poly (vinylidene fluoride) (PVDF) is attractive in energy conversion applications between electrical and mechanical forms because of its low cost, high flexibility, and biocompatibility [4,5]. The piezoelectric property of PVDF has been utilized in various device applications, such as strain sensors [6], mechanical actuators [7], energy harvesters [8], and artificial muscles [9]. PVDF consists of four crystalline phases, α, β, γ, and δ. Among the three polar phases, the β-phase has the largest spontaneous polarization (p) per unit cell that has all the dipolar moments pointing to the same direction and has the best piezoelectric responses, and the non-polar α-phase is the most stable phase [10]. Polymer nanofibers are mostly produced by electrospinning technology. Procedures for subsequent stretching and poling can be eliminated via electrospinning [11]. Electrospinning allows for the facile production of continuous fibers with diameters ranging from tens of nanometers to several micrometers [12]. Many researchers used this method to improve PVDF properties by converting the α phase into the β phase, which is responsible for the piezoelectric properties of PVDF [13,14,15].

Because of the unique properties of PVDF nanofibers and its application in power generation, in addition to some modification on solution parameters and processing conditions [16,17,18], researchers have been investigating the addition of additives such as inorganic salts (NaCl, KBr, LiCl, KCl) [19,20,21], piezoceramic nanoparticles [22,23], organic salts, and carbon nanotubes (CNT) [24] on fiber morphology for polymer solutions. Abolhasani et al. demonstrated the application of graphene-PVDF composite nanofibers for portable self-powering electronic devices. The addition of 0.1 wt % of graphene increased F(β) from 77% to 83%. However, further addition of graphene decreased the F(β) to 75%. Open-circuit voltage of PVDF nanofiber was 3.8 V, while the addition of 0.1% graphene increased this value to 7.9 V [25]. NaNbO3 nanorods help in the alignment of the electric dipoles in PVDF and, because of the inherent piezoelectric property, contribute to the overall piezoelectric property [26]. Zeng et al. embedded the PVDF/NaNbO3 as nanofiber nonwoven fabric sandwiched between two electrically conducting knitted fabric electrodes, which consistently produce a peak open-circuit voltage of 3.4 V and a peak current of 4.4 μA in a cyclic compression test [27]. Polymers have been found to exhibit high breakdown strength along with high energy density, while the fillers, especially ceramics, have high dielectric constant. The combination of both provides enhanced dielectric properties depending on the type and nature of polymer matrices as well as fillers [28]. Adding 5 wt % of BaTiO3 had the highest β-phase crystallinity for nanocomposite inkjet inks [29]. The PVDF-HFP (hexafluoropropylene)/PVDF (core/shell) containing ZnO and TiO2 nanoparticles exhibits a maximum output voltage of 14 V [30]. With regards to the use of inorganic salts, Arayanarakul et al. used inorganic salt of various types (NaCl, LiCl, KCl, MgCl2, and CaCl2) for poly(ethylene oxide) (PEO) solution in the electrospinning process. Among the various examined inorganic salts, only NaCl by an increase in the conductivity and a decrease in the viscosity was able to improve the electro-spinnability of the 4% (w/v) PEO solution [31]. Smaller inorganic salts like LiCl and NaCl, with their relatively smaller ionic radii, higher charge density, and thus higher mobility under applied external electric fields, are expected to have a pronounced effect on the resultant fibers [32]. Our previous research work showed that adding 0.00133 wt % LiCl to the PVDF solution increased the elongation at break of samples four times more than pure PVDF ones. The β phase formation and output voltage increased up to 90% and 900 mV, respectively [33].

The effect of PVDF fiber thickness on the electroactive phase formation showed an increase in the β/α ratio from 1.06 for the thin fiber (≈50 nm) to 1.39 for the thicker fiber (≈1500 nm) [34]. In piezoelectric nanogenerator devices, because the total dipole moment is proportional to the quantity of piezoelectric nanofibers, the thickness of the electrospun web that could be modulated by the spinning time is a crucial factor for determining the piezoelectric performance [35]. Gheibi et al. presented a one-step fabrication process of a pure piezoelectric PVDF nanofiber web that can be used to convert mechanical energy to electrical power; the results showed that increasing the thickness of nanofiber webs can lead to a decrease in the output voltage of nanogenerators [36]. Although significant studies have been done for the evaluation of piezoelectric output, as mentioned above, the effect of temperature on the electrospun PVDF nanogenerators performance is rarely addressed. In most cases, the effect of temperature variation is studied on PZT (lead zirconate titanate) films because of their wide applications [37,38]. As material constants of a piezoelectric material strongly depend on temperature, the performance of the piezoelectric device may vary significantly. In addition, investigation of a piezoelectric material that can work robustly at a higher temperature is required to maximize the potential for applications [39]. A PVDF piezoelectric nanofibrous film was made by the electrospinning technique and exposed to annealing as a post processing treatment at 40, 70, 100, and 130 °C for 4 h in an air-flow oven. The enhanced voltage output of the nanogenerator is recognised to be the collaborative involvement of the electrospinning and annealing treatment, which could improve the piezoelectric response of the annealed film [40]. Shaik et al. realized 87% of β-phase with 20 wt % PVDF solution spinning coated on the substrate at 9000 rpm and 100 °C annealing temperature and further proved that the annealing temperature can affect the β-phase content in spin-coated PVDF films, particularly at a higher temperature around 95 °C under lower rotation speed [41].

Because of the wide applications of piezoelectric materials, characterization and optimization of polymer-based nanogenerators is important for future applications. Therefore, based on the weak piezoelectric response of the electrospun pure PVDF web, in the present study, LiCl was introduced as an additive and the optimized concentration was used to improve the formation of the β phase, which is responsible for the piezoelectric properties of PVDF. The performance of the PVDF/LiCl nanogenerator was compared with different web thicknesses during impact and vibration tests. Moreover, the piezoelectric response of the nanogenerator was evaluated during temperature variations.

## 2. Experimental

### 2.1. Materials

PVDF pellets were provided by Sigma-Aldrich (Lyon, France) with molecular weight of 534,000 (g/mol^−1^). The solvents used in this work were N-Ndimethyl formamide (DMF, Merck Chem. Co., Hohenbrunn, Germany) and acetone (Merck Chem. Co., Hohenbrunn, Germany). Lithium chloride was used as inorganic salt additive (LiCl, Merck Chem. Co., Hohenbrunn, Germany). All the materials were used without further purification.

### 2.2. Methods

#### 2.2.1. Preparation of Spinning Solutions

In this research, dimethyl formamide (DMF)/acetone was used as solvent with a ratio of 4/6 (*v/v*) for PVDF/solvent concentration of 16% (*w/w*). PVDF was initially dissolved in DMF and the PVDF/DMF solution was stirred for 1 h at 60 °C. LiCl was used after drying in a vacuum oven for approximately 24 h at 120 °C. LiCl with a concentration of 0.00133 wt % was added into the solution. Finally, acetone was added, and the solution was stirred again for 24 h at room temperature.

#### 2.2.2. Preparation of the PVDF/LiCl Nanofibers Web

The electrospinning setup used in this study consisted of a 1.0 mL plastic syringe tipped with a 22-gauge stainless steel needle (e.d. = 0.7 mm, i.d. = 0.4 mm) and a high-voltage supply (Gamma High Voltage Research, Tehran, Iran). The needle was connected to the high-voltage supply, which could generate positive direct current voltages up to 40 kV. The distance between the needle tip and the ground electrode was adjusted to 20 cm. The positive voltage applied to the polymer solution was set at 20 kV. The PVDF solution was delivered via a syringe pump (KD Scientific, Holliston, MA, USA). This pump controlled the mass flow rate at 0.3 mL/h. An aluminum sheet was used as the collector plate. The entire electrospinning fiber formation process was carried out at room temperature.

#### 2.2.3. Fabrication of Power Generator Samples

**Figure 1:**
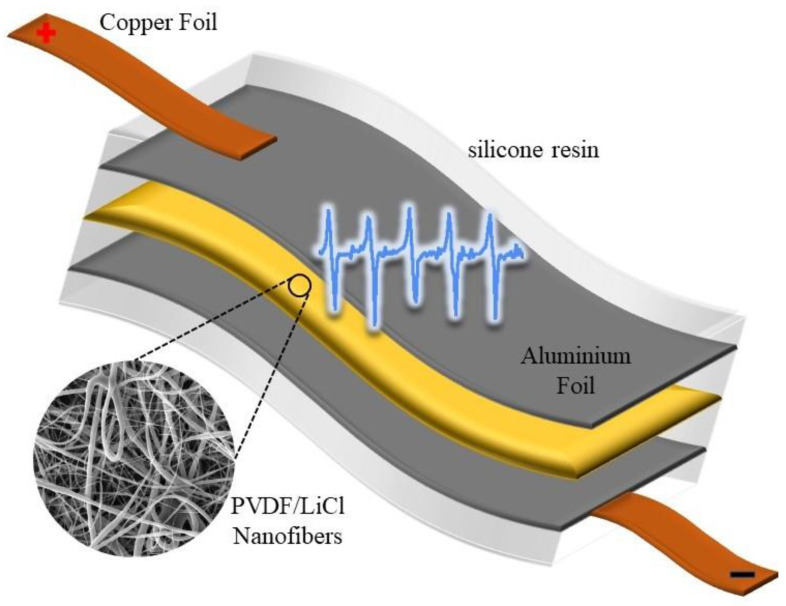
Schematic structure of the polyvinyledene fluoride (PVDF)/LiCl nanogenerator device.

In order to fabricate a nanofibrous power generator, the aluminum foil was used as collector plate and the electrospinning process was carried out on this substrate. A rectangular piece (3 × 1.5 cm) of PVDF/LiCl web was used as an active layer and sandwiched between two aluminum foils. Two copper strips by silver paste were set on both sides of the aluminum foils because of their good conductivity and wire soldered onto them to connect to the data acquisition board. The entire nanogenerator device was fully covered with silicone resin to enhance mechanical robustness and protect it from dust and water (Figure 1). For more information about sample preparation, refer to our recent publication [10].

#### 2.2.4. Measuring Output Voltage of PVDF/LiCl Nanogenerator

**Figure 2:**
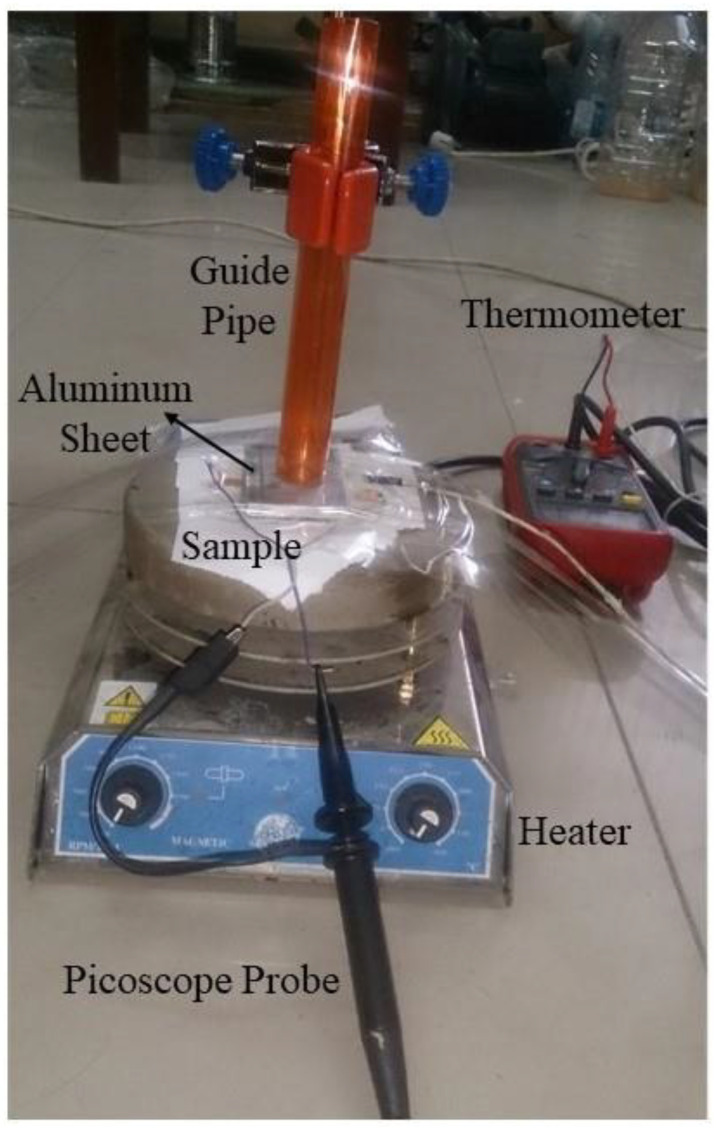
Experimental setup of the impact test during temperature variations.

The excitation of the piezoelectric package was accomplished by two methods. One was using the base motion of a shaker at 200 HZ and the other was the drop weight impact test on the nanogenerator and tracing the falling trend (Figure 2). A steel ball (15 mm in diameter, 16.5 g in weight) was dropped from different heights through a guide pipe and applying impact to the PVDF/LiCl nanogenerator device. The electrical response of the piezoelectric to the applied stress was recorded by connecting the electrodes to an oscilloscope (Pico Scope 4224, St. Neots, UK). To protect samples from potential damage caused by repetitive impacts as well as to excite and obtain voltage measurements from large active areas of samples, an aluminum sheet (thickness of 5 mm) was placed on samples during the impact tests and the whole system (samples and aluminum sheet) was fixed with tape to the wooden surface. Two electrodes are used on the top and bottom surface of the electrospun web. The voltage difference between the two adjacent electrodes was thereby induced owing to this separation of charge. The electrodes’ types enhance the power output of the nanogenerator.

#### 2.2.5. Measuring Output Voltage During Temperature Variations

Different parameters affect the output voltage of nanogenerators such as materials, additives, package fabrication, substrate, and excitation method, but very little is known about the effect of temperature on nanogenerator performance. The aim of this paper is to investigate the effect of temperature variations on the output voltage of the nanogenerator devices from different dropping heights. The experimental condition was like the impact test described above, but the temperature was varied from 30 to 90 °C and a thermometer was used to measure the samples’ temperature. The hotplate temperature was set for 2 min prior to measuring the temperature of the nanogenerator device.

## 3. Results and Discussion

### 3.1. Effect of LiCl on β Phase Formation 

According to the Fourier-transform infrared (FTIR) results, the β-phase content increased from 84% to 94% by adding 0.00133% LiCl to the pure PVDF. The Differential scanning calorimetry (DSC) analysis indicates that more crystals were formed during the electrospinning of PVDF/LiCl nanofibers. In the X-Ray Diffraction (XRD) analysis, the α-phase peak is reduced in intensity for electrospun PVDF/LiCl (277.68) compared with the electrospun PVDF (286.18). Hence, this may suggest that most of the crystal phase in electrospun PVDF was changed to β-phase by adding LiCl to the polymer solution, whereby the β-phase crystal structure becomes dominant with some transitions or mixtures of α and β phases. For more information, refer to our recent publication [10].

### 3.2. Effect of LiCl in Piezoelectric Response

**Figure 3:**
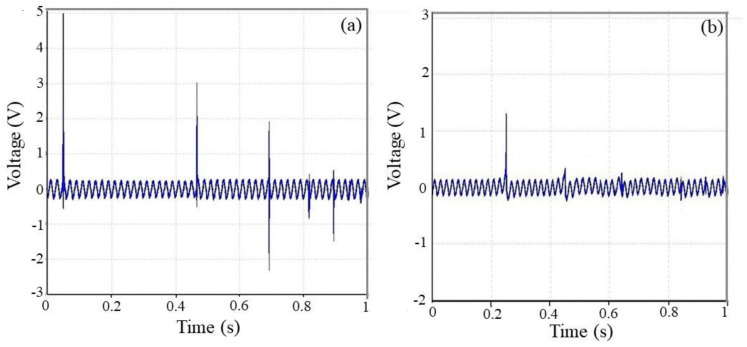
Piezoelectric response of nanogenerators, excited by the drop weight impact test on the electrospun web of in 250 μm thickness: (**a**) PVDF/LiCl (0.00133 wt %) and (**b**) pure PVDF.

Here, in order to study the effect of LiCl additive on the output voltage of electrospun PVDF fiber and evaluate its piezoelectric properties, the drop of small ball from the same height was used to apply a periodic dynamic loading on top of the samples (size: 3 × 1.5 cm). As can easily be seen from Figure 3, the maximum peak of output voltage for the PVDF sample with and without LiCl is 5 and 1.3 V, sequentially. The results confirmed that, because of the formation of the β phase, LiCl improves the piezoelectric response of piezoelectric properties of PVDF without the need to any post treatments for the nanogenerator device.

To ensure that the voltage output only arises from the electrospun PVDF nanofiber web, a paper layer was used instead of the nanofiber web between the two electrodes, and then the output voltage was measured. The results showed no signal voltage output from the paper, as expected. The key to prepare high piezoelectricity of the PVDF polymer is inducing β-phase formation, however, untreated PVDF cannot have β-crystalline without electrical or mechanical poling processes [42]. Applying a high electrical field during the electrospinning processes helps to orient the polar dipoles in the PVDF structure. This process can be analogous to the electrical poling process. Therefore, the electrospinning process promotes the formation of the β-phase without any need for post-treatment processes including electric poling and mechanical stretching [14]. However, the poling process was used as a post treatment to increase the voltage response of pure PVDF electrospun web by some researchers [36]. The addition of additives or fillers at a certain range of concentrations leads to the increase in the electroactive β-phase, as does the piezoelectric property. Organic and inorganic fillers have been used to modify the functionality of the PVDF polymer [13]. The optimization of LiCl concentration and improvement evidence of the β phase formation by adding LiCl compressively was discussed in our previous work, where the best result for salt-concentration was obtained from 0.00133 wt.% LiCl. It was found that the β-phase content increases from 84% for pure PVDF nanofiber and reaches a maximum value of 94% for PVDF/LiCl nanofiber containing 0.00133 wt % of LiCl [10]. The addition of a small amount of LiCl into the PVDF solution resulted in a reduction in the average diameter of the as-spun fibers. Inspired by the ion-dipole interactions between ionic salt and polymer, herein, by adding soluble LiCl salt to the polymer solution, which dissociates into equal numbers of positive and negative ions, the electrical conductivity of the solution by increasing the number of ions per unit volume is increased [10,33]. During electrospinning, when an external electrical field was applied to the solution, the positive and negative ions in the polymer fluid tend to move in the opposite direction. Negative ions are forced toward the positive electrode, and positive ions are forced toward the negative electrode, the result of which is stretching and elongation, as mentioned above [20].

### 3.3. Effect of Electrospun Web Thickness on Piezoelectric Response

**Figure 4:**
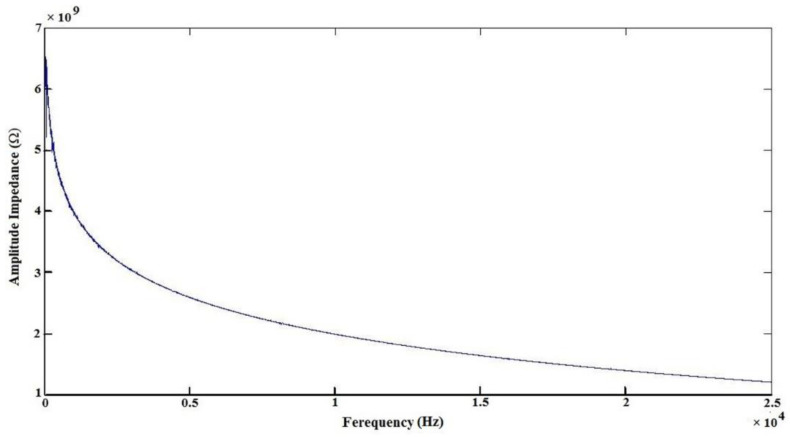
The experimental amplitude impedance of electrospun PVDF nanofibers mats as a function of frequency at room temperature.

Sensors based on piezoelectric materials are the most suitable for applications under time-dependent mechanical excitations [43]. In this regard, sensors are evaluated from different perspectives, one of which is the thickness of the sensor. The nanogenerators’ configuration could be varied in terms of its thickness by modulating the electrospinning time. The optimized substrate to piezoelectric layer thickness ratio showed that the substrate thickness affects the performance of the energy harvester and, for a low thickness ratio, structure 2 performs better than the other structures considered [44]. Gheibi et al. showed that increasing the thickness of nanofiber pure PVDF membranes leads to a decrease in the output voltage of the nanogenerator [45]. The choice of substrate type and web thickness is important in optimizing the sensor geometry and the sensor performance for the specific application. One of the main privileges of the nanofiber web-based pressure sensor or generator compared with the film form and ceramic-based piezoelectric materials is that the expectable piezoelectric effect is much higher because of the high compressibility associated with the specific range of thickness change at the same pressure. Hence, this paper attempts to introduce a nanogenerator package with more bending comfort and high output voltage, without need for post treatment (electrical poling) and repeatability of response and sensitivity. Therefore, after optimization of LiCl concentration, the effects of web thickness on piezoelectric response in the following discussions are assessed. The amplitude impedance obtained via applying frequency using an impedance analyzer is shown in Figure 4. This behavior indicates that the piezoelectric device (consisting of a PVDF/LiCl electrospun nanofiber web and two aluminum electrodes) has an acceptable capacitance property.

#### 3.3.1. Vibration Test

**Figure 5:**
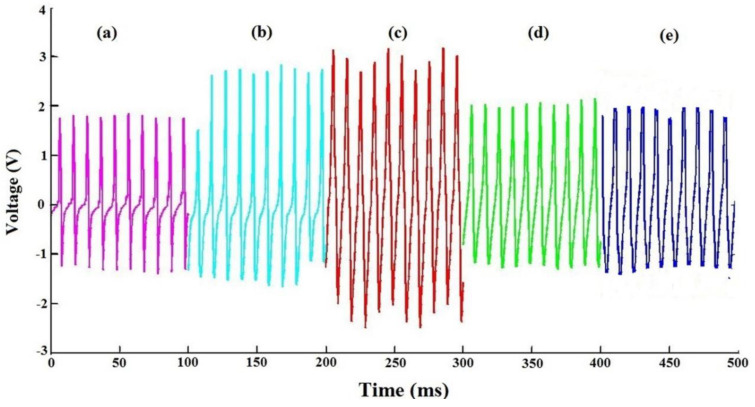
Piezoelectric response of the electrospun PVDF/LiCl nanogenerator obtained using the base motion of a shaker at 200 HZ frequency for different web thicknesses: (**a**) 150, (**b**) 250, (**c**) 350, (**d**) 450, and (**e**) 550 μm.

Figure 5 demonstrates the results obtained by actuating the electrospun PVDF/LiCl nanofiber web at a frequency of 200 Hz. The enhancement in the strain sensing ability of the electrospun PVDF/LiCl nanofiber web was attributed to the combined effect of the piezoelectric properties of the electrospun PVDF polymer and LiCl as salt additive. The experiments performed here were repeated several times in order to assure repeatability of the results. The measurements showed a periodic alternation of positive and negative output peaks, corresponding to the application and release of the vibration test, respectively. The range of voltage outputs is 1.8–3.1 V. The output voltages increased with increasing electrospun web thickness. Persano et al. also have the same results for aligned arrays of nanofibers of poly (vinylidenefluoride-co-trifluoroethylene), but generating for a maximum voltage of 1.2 V for a thickness of 225 μm [46].

Kanda et al. evaluated the influence of parasitic capacitance on the output voltage for series-connected thin-film piezoelectric devices. In the design of devices that employ output-voltage multiplication, the estimation of the optimum number of series connections and the effect of parasitic capacitances are important [47]. Here, as obtained from Figure 5, there is an optimum value for electrospun PVDF/LiCl web thickness, after increasing the thickness up to 350 μm, faced with a reduction in output voltage. The same results were obtained for the electrospun web of pure PVDF with a thickness of around 310 μm [45].

**Figure 6:**
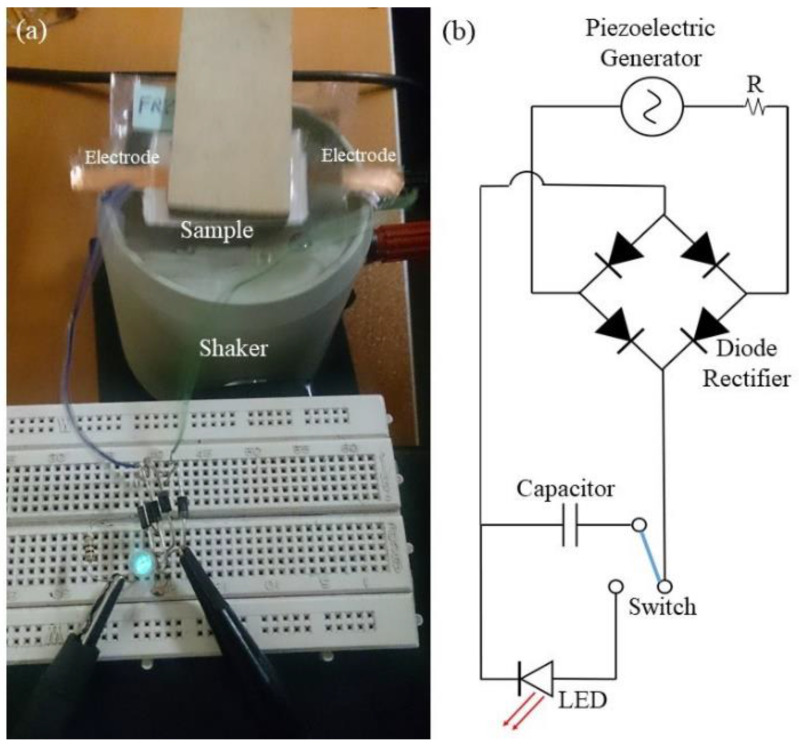
(**a**) A snapshot of flashing LEDs during excitation by shaker at 200 Hz, (**b**) schematic diagram of the prototype energy-harvesting circuit.

To explain the relationship between the output voltage and the electrospun web thickness, one needs to understand that the voltage output of a piezoelectric material is a function of its capacitance. If the piezoelectric layer is very thin, there would be very high capacitance (C) and low charge (Q). Thus, from $V=\frac{Q}{C}$, the voltage output would be low. The other extreme is when the piezoelectric material is very thick, such that the deflection of the structure is significantly reduced, and hence very little charge is generated. Hence, the optimum voltage is obtained at values of the thickness between these two extremes [36]. In the optimum condition of LiCl concentration, electrospinning parameters, and web thickness (350 μm), a light-emitting diode (LED) can be lit by vibrating excitation of the nanogenerator at 200 Hz (Figure 6).

**Figure 7:**
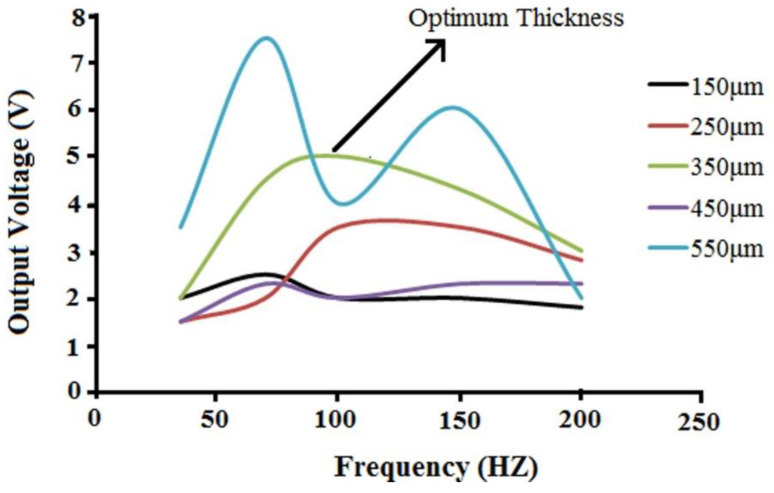
Effect of frequency on the voltage output for various electrospun PVDF/LiCl web thicknesses.

Studies show that the voltage outputs versus frequency for PZT and PVDF generators have been smoothed with a polynomial trend line. With an increase in frequency, the output voltage increased up to an optimum value [48]. By increasing the frequency, the electrons in the external circuit have a shorter time to balance the piezoelectric potential, and this will lead to a larger current. As the external voltage is the product of the current and external resistance, the voltage will get bigger correspondingly. As can be seen from Figure 7, this trend is just followed by an optimum thickness value of PVDF/LiCl nanogenerator at 350 μm. Other trends do not follow this polynomial trend because they are not in optimum thickness conditions.

#### 3.3.2. Impact Test

**Figure 8:**
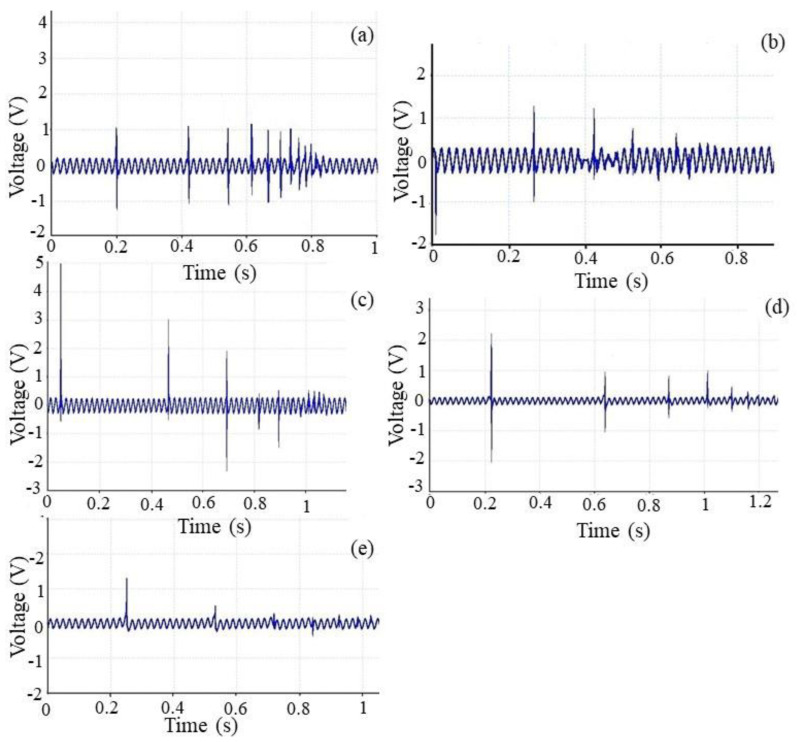
Representative piezoelectric response of PVDF/LiCl nanogenerators. Excitation device obtained from a ball falling down for different web thicknesses: (**a**)150 μm, (**b**) 250 μm, (**c**) 350 μm, (**d**) 450 μm, and (**e**) 550 μm.

When forces are applied on an electrospun piezoelectric web, voltage is built up on both sides of it because of the piezoelectric effect. This unique property can be utilized to generate/harvest electricity from environmental vibration such as human motion for smart textiles and medical applications. The impact experiments involve releasing a mass (ball weighing 16.5 g) from a fixed height maintained for the electrospun PVDF/LiCl web. The ball was allowed to fall freely from a fixed height and the output voltages were measured versus time. It was shown that 11.9 g impact mass yielded larger power output voltages compared with those by the smaller 4.9 g mass for both ceramic and polymer piezoelectric materials [47]. Accordingly, in this paper, an attempt is made to choose a ball weight in this range (16.5 g). The free falling object impacted the nanogenerator as close to the center as possible. One end of the Picoscope was connected to a PC and the other end to positive and negative polarities on the sample to record the output voltage versus time. The Picoscope software was configured to display the graph with the time frame set from 0 to 1000 ms. The mass was dropped onto the sample one time in this time frame. Figure 8 shows a typical output spectrum for different thicknesses of the electrospun PVDF/LiCl samples when subjected to the impact test. The main explanation of the results is that the intensity of polarization in the PVDF/LiCl electrospun web will be changed when it suffers a falling impact. According to the experiments mentioned above, the PVDF/LiCl electrospun nanogenerator could respond to the impact test and transform the mechanical energy into an electrical signal, but there were differences in their response signals when the web thicknesses were changed. Jia et al. evaluated the dependence of the PVDF sensors’ response (100 μm PVDF film) on the elasticity of the supporting materials (desk, rubber, and sponge) by free falls of different weights from different heights. The results showed that the desk supported PVDF sensor could convert the impact energy into a relatively bigger Vpp than the same sensor supported by rubber (82.26 V/J) or sponge (about 96.62 V/J), but the desk supported sensor was responsive enough to measure the impact intensity, because the values of Vpp were almost constant when the impact energies were changed [49]. Therefore, in this paper, a rigid surface is chosen as the backstop, which is illustrated in Figure 2. As obtained from Figure 8a–e, during the impact test, the trend of maximum output voltage peaks increases by increasing the thickness until optimum value is established and then, after the optimum thickness (350 μm), a descending trend is faced.

It was found that the biggest sensitivity of the voltage response is about 208.3 V/J for the optimum thickness. The important thing to note here is to consider damping of the system. For a thinner web (stiff substrate), the ball falling down appears to move fast and shows more dapping, and the distance between the peaks is smaller, but for thicker webs (softer substrate), the distances between peaks are greater, so this package is also sensitive to thickness.

### 3.4. Effect of Temperature Variation on the Piezoelectric Response

**Figure 9:**
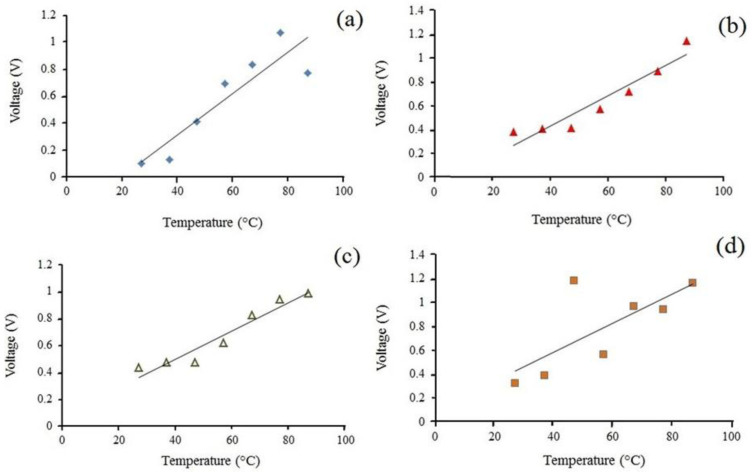
Comparison of output voltage for PVDF/LiCl electrospun webs when subjected to the impact test from different dropping height of (**a**) 15 cm, (**b**) 20 cm, (**c**) 25 cm, and (**d**) 30 cm at various temperatures.

For the effect of temperature, the temperature was increased from 30 °C to 90 °C while applying pressure, and it was found that the signal of the nanogenerator increased linearly with temperature, as shown in Figure 9. The Curie temperature—when the piezoelectric response of PVDF polymers vanishes—is about 165–170 °C [50]. The change in temperature alters the position of the atoms within the crystal structure, and as such, the polarization of the material will be changed; this polarization change gives rise to a voltage across the crystal [51]. The increase in voltage due to increased temperature could be associated with the enhanced excitation of molecules within PVDF material, resulting in faster electron movements as the energy of the material is increased at higher temperatures [47]. Moreover, as the temperature of the heat source increases, the dielectric constant of PVDF increases, which leads to an increment in output voltage [38]. When the temperature was raised from 30 °C to 90 °C, the nanogenerator showed the sensitivity of 0.16%/°C under 100 Pa pressure and 0.06%/°C under 220 Pa pressure. The trends of output voltage at elevated temperatures for all dropping heights are incremental, but these trends, as can be seen from Figure 9, are more uniform for heights of 20 and 25 cm. By increasing the temperature, electrons move faster; besides, when a force applied from a higher dropping height, it leads to disarranged electron movement and to interference in piezoelectric performance, which can be a reason for this ununiformed increment trend for a higher height. The temperature dependence of electrospun PVDF/LiCl webs’ output voltage is completely opposite to the behavior of the PZT piezoelectric membrane reported by previous studies [48]. This preference can be used for a variety of applications in areas in which they will experience harsh environmental conditions with extreme temperature fluctuations, such as aerospace and automobile tyres.

## 4. Conclusions

We have demonstrated a one-step fabrication of the energy harvesting textiles based on piezoelectric PVDF electrospun nanofibres that enables the conversion of mechanical energy to electrical power. We have developed a lightweight and flexible nanogenerator through an electrospinning process to create a self-powered temperature sensor. The influence of LiCl in piezoelectric response, which is presented in this article, can be a cost-effective and easy method to increase the output voltage of nanogenerators. The effect of the as-prepared electrospun PVDF/LiCl nanofiber web thicknesses on output voltage was evaluated, the optimum value base on the output voltage of which is found to be 350 μm, with the highest sensitivity of ~208.3 V/J. Furthermore, the temperature dependence of the nanogenerator shows that the output voltage was increased linearly in the range of 30–90 °C. The developed nanogenerators are likely to find applications such as wearable electronic systems, safety monitoring, smart sensors, and medical diagnostics.
